# *Cinnamomum cassia* Presl: A Review of Its Traditional Uses, Phytochemistry, Pharmacology and Toxicology

**DOI:** 10.3390/molecules24193473

**Published:** 2019-09-25

**Authors:** Chunling Zhang, Linhong Fan, Shunming Fan, Jiaqi Wang, Ting Luo, Yu Tang, Zhimin Chen, Lingying Yu

**Affiliations:** School of Pharmacy, Chengdu University of Traditional Chinese Medicine, Chengdu 611137, China; zhangchunling1997@163.com (C.Z.); fanlinhong1996@163.com (L.F.); fanshunming@stu.cdutcm.cn (S.F.); kikiAPTX@163.com (J.W.); lt530794033@163.com (T.L.); Ty593812828@163.com (Y.T.)

**Keywords:** *Cinnamomum cassia* Presl, traditional uses, phytochemistry, pharmacology, toxicology

## Abstract

*Cinnamomum cassia* Presl is a tropical aromatic evergreen tree of the Lauraceae family, commonly used in traditional Chinese medicine. It is also a traditional spice, widely used around the world. This paper summarizes the achievements of modern research on *C. cassia*, including the traditional uses, phytochemistry, pharmacology and toxicology. In addition, this review also discusses some significant issues and the potential direction of future *C. cassia* research. More than 160 chemicals have been separated and identified from *C. cassia*. The main constituents of *C. cassia* are terpenoids, phenylpropanoids, glycosides, etc. Modern studies have confirmed that *C. cassia* has a wide range of pharmacological effects, including antitumour, anti-inflammatory and analgesic, anti-diabetic and anti-obesity, antibacterial and antiviral, cardiovascular protective, cytoprotective, neuroprotective, immunoregulatory effects, anti-tyrosinase activity and other effects. However, the modern studies of *C. cassia* are still not complete and more in-depth investigations need to be conducted in alimentotherapy, health product, toxicity and side effects, and more bioactive components and potential pharmacological effects need to be explored in the future.

## 1. Introduction

**Figure 1:**
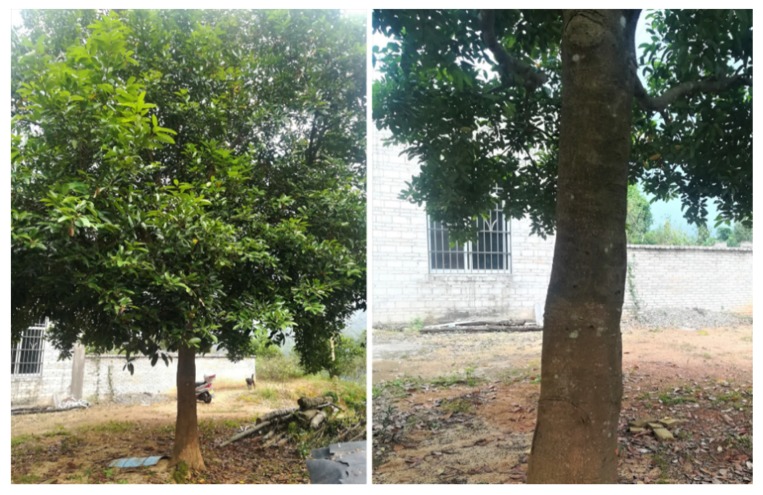
*Cinnamomum cassia* Presl.

*Cinnamomum cassia* Presl is an aromatic tree species belonging to the Lauraceae family. From the bark of its young branches, cinnamon is obtained, which is widely used all around the world for its fragrance and spicy flavor (Figure 1). It can be used not only as a daily condiment, but also as a raw material for medical products, and has high economic value. *Cinnamomum cassia* Presl is distributed in China, India, Vietnam, Indonesia and other countries; In China, the producing areas are mainly concentrated in Guangxi, Guangdong, Fujian and Hainan provinces. Cinnamomi cortex is the bark of *C. cassia*, which is often used as a seasoning and spices in the West. For instance, in America, Cinnamomi cortex is used as a food supplements, as a coumarin source of [1]. In Asia, Cinnamomi cortex is usually used as a drug. Cinnamomi cortex is a common traditional Chinese medicine in China. Since 1963, Cinnamomi cortex has been listed in the Pharmacopoeia of the People′s Republic of China (CH.P), and there are more than 500 formulas containing Cinnamomi cortex used to treat various diseases, such as cardiovascular disease, chronic gastrointestinal disease, gynecological disorders and inflammatory disease [2,3,4]. Currently, a lot of studies have been done on the pharmacological and phytochemical of *C. cassia*, and more than 160 chemicals have been separated and identified from *C. cassia*. More and more studies have confirmed that *C. cassia* has a wide range of pharmacological effects, including antitumour, anti-inflammatory and analgesic, anti-diabetic and anti-obesity, antibacterial and antiviral, cardiovascular protective, cytoprotective, neuroprotective, immunoregulatory effects, anti-tyrosinase activity and other effects [3,4]. So far, the CH.P still recognizes Cinnamomi cortex as a common traditional Chinese medicine, and the content of cinnamaldehyde is used as an evaluation index for evaluating the quality of Cinnamomi cortex.

## 2. Traditional Usages

As a traditional Chinese medicine *Cinnamomum cassia* Presl has a wide range of pharmacological activities and a long history of use as a drug. The earliest medicinal history of this plant was recorded in the *Shennong Bencao Jing*, which is the earliest and most important encyclopaedia of traditional Chinese medicine in the Eastern Han Dynasty (25–220 AD). In this classic, *C. cassia* was used for treating arthritis. In *Mingyi Bielu*, the function of *C. cassia* was analgesic. In *Yaoxing Lun*, which is another known traditional Chinese medicine classic, *C. cassia* was used for treating bellyaches and dysmenorrhea. In addition, *C. cassia* was also recorded in other famous traditional Chinese medicine books, such as *Tangye Bencao*, *Bencao Gangmu*, *Bencao Jingshu*, *Bencao Huiyan,* etc. Nowadays, *C. cassia* has become a common traditional Chinese medicine for treating nephropathy, dysmenorrhea, menoxenia and diabetes [5,6]. In order to be applied to clinic better, various dosage forms, such as pills, capsules, granules, oral liquid and so on, have been developed (Table 1).

## 3. Phytochemistry

**Figure 2:**
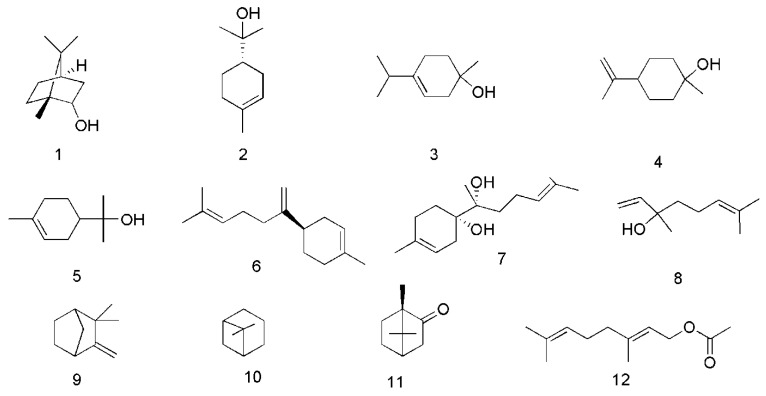
Chemical structures of the monoterpenes in *C. cassia*.

**Figure 3:**
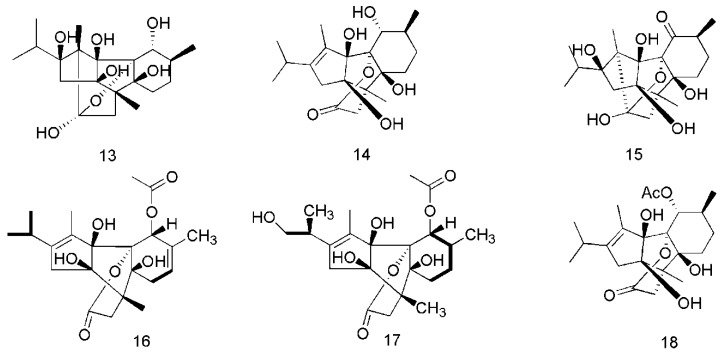

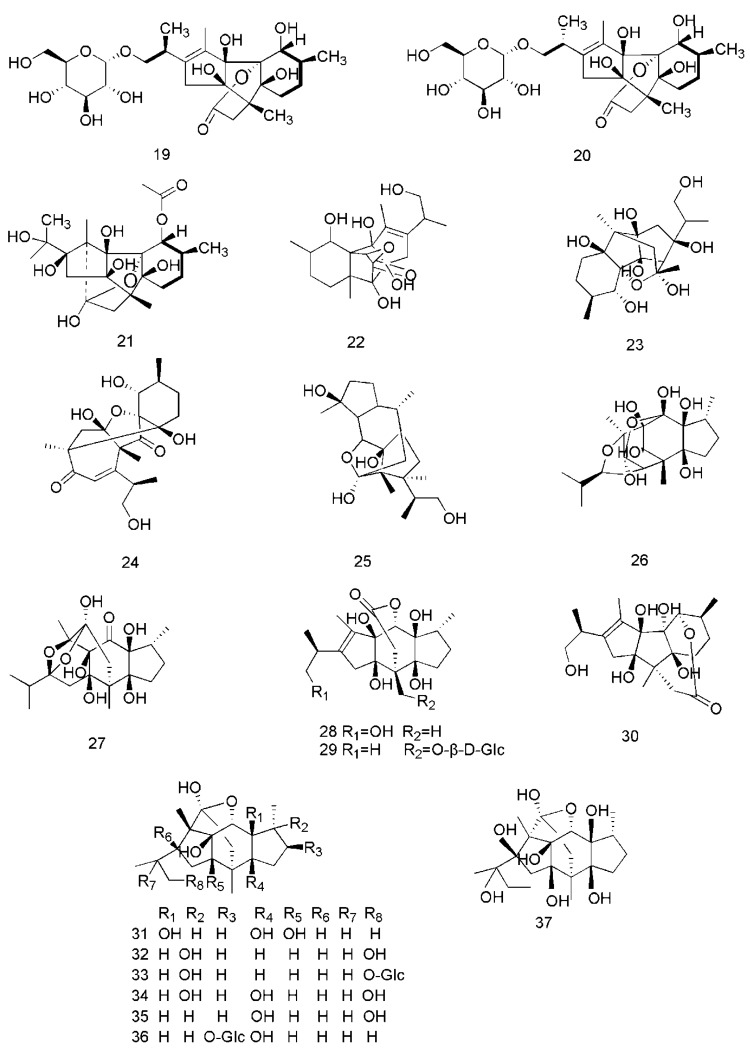
Chemical structures of the diterpenoids in *C. cassia*.

**Figure 4:**
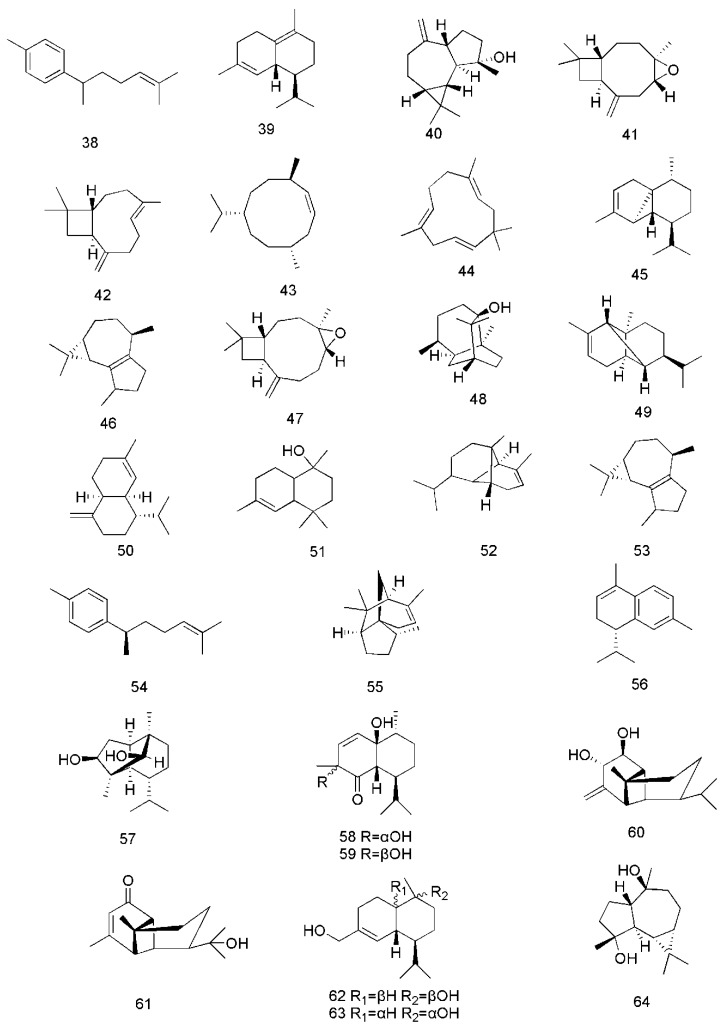
Chemical structures of the sesquiterpenoids in *C. cassia*.

**Figure 5:**
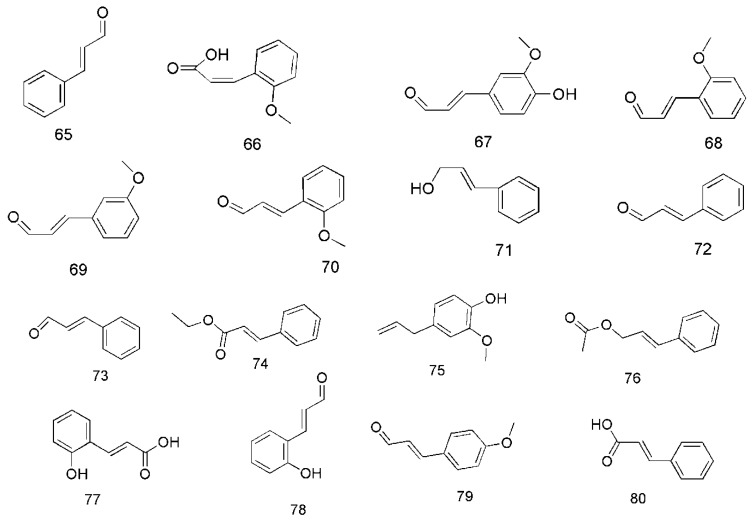
Chemical structures of the phenylpropanoids in *C. cassia*.

**Figure 6:**
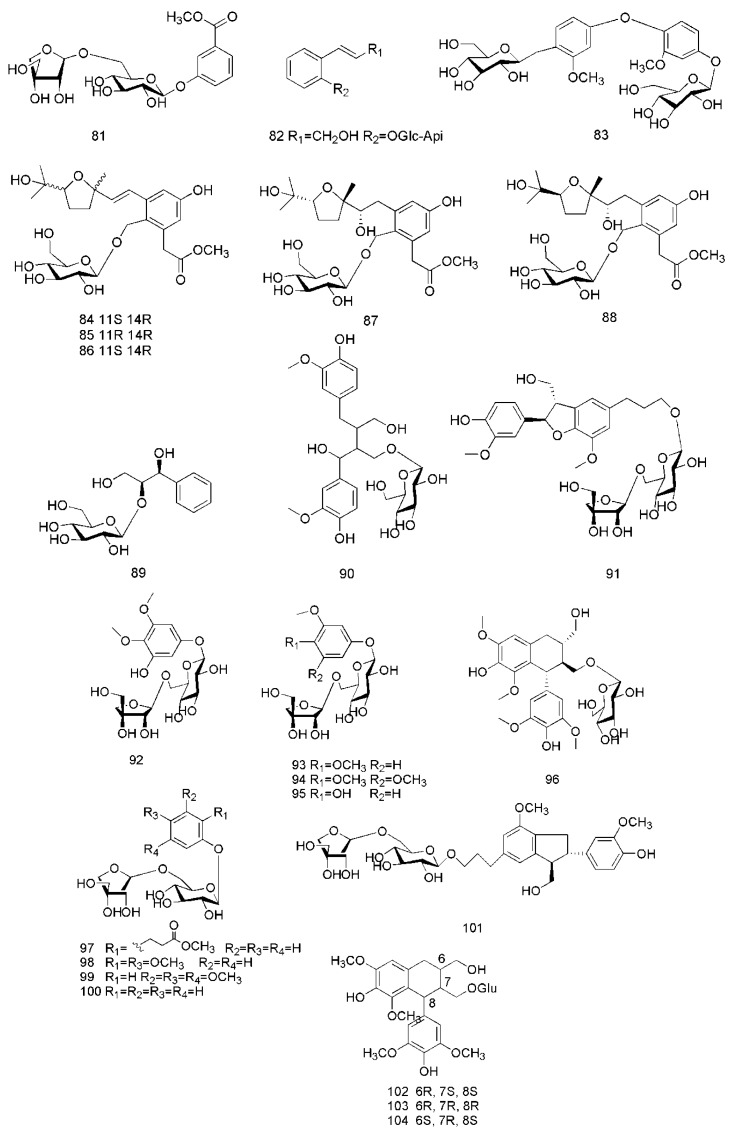
Chemical structures of the glycosides in *C. cassia*.

**Figure 7:**
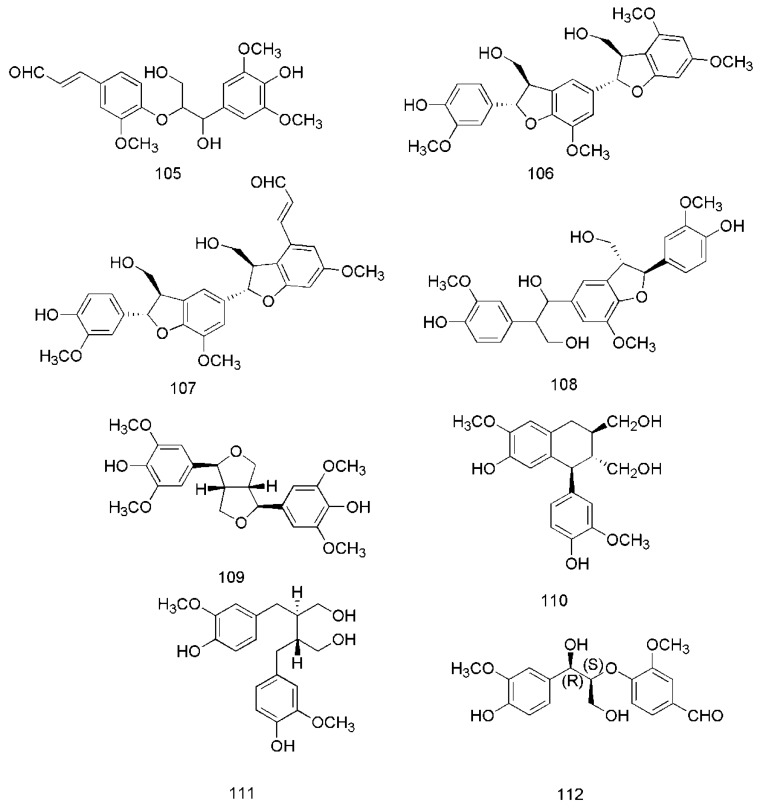

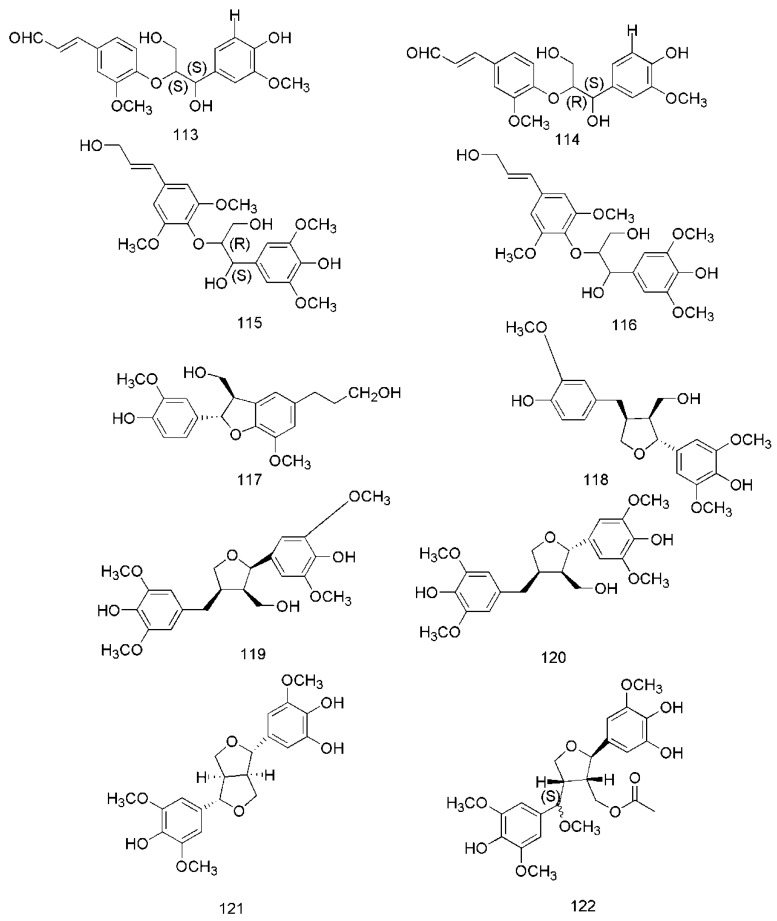

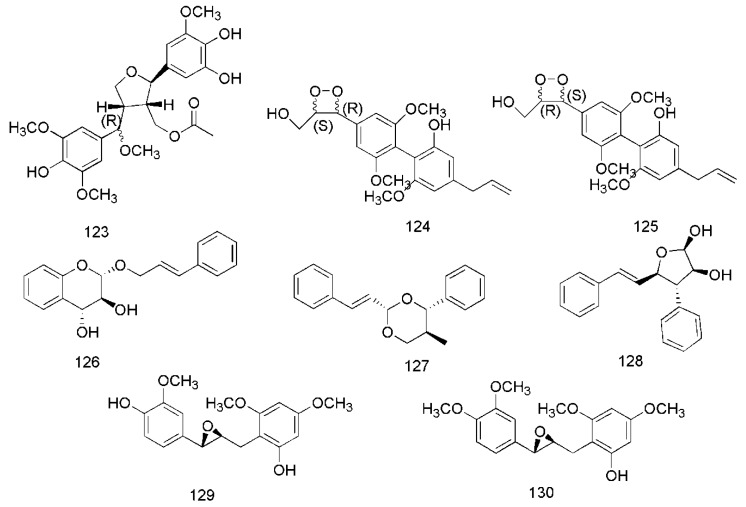
Chemical structures of the lignans in *C. cassia*.

**Figure 8:**
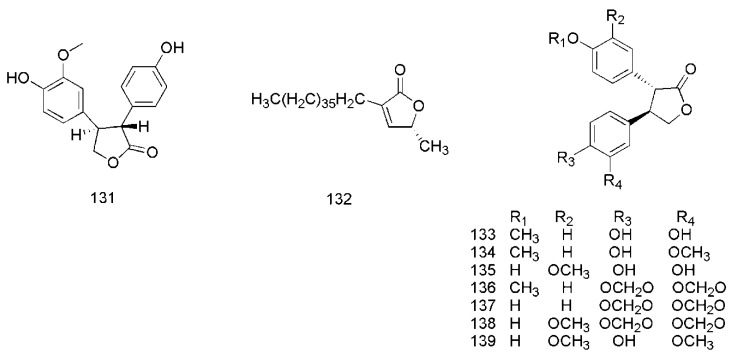
Chemical structures of the lactones in *C. cassia*.

**Figure 9:**
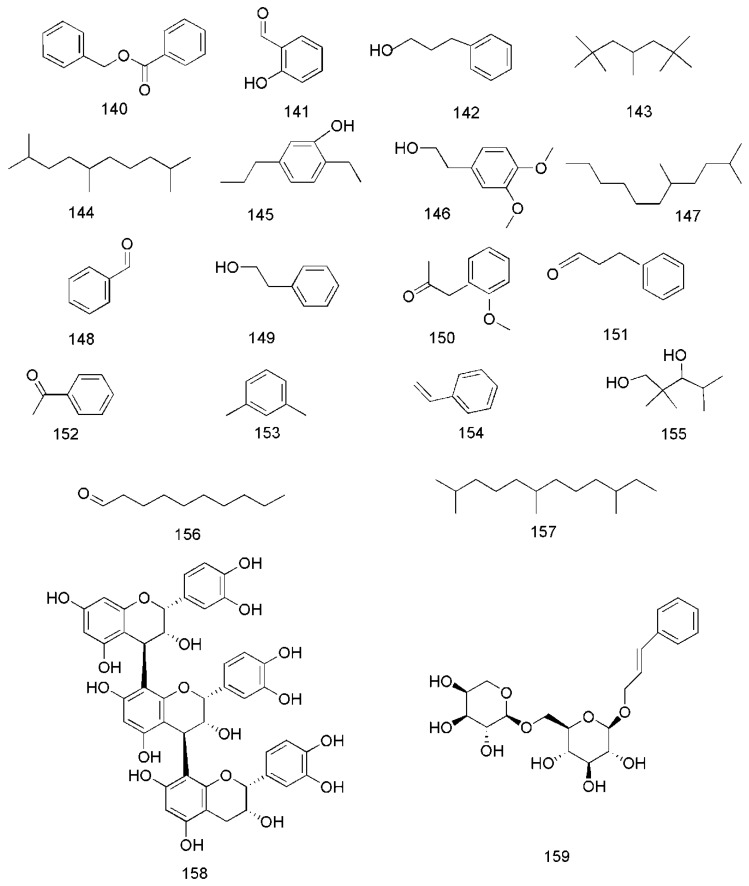

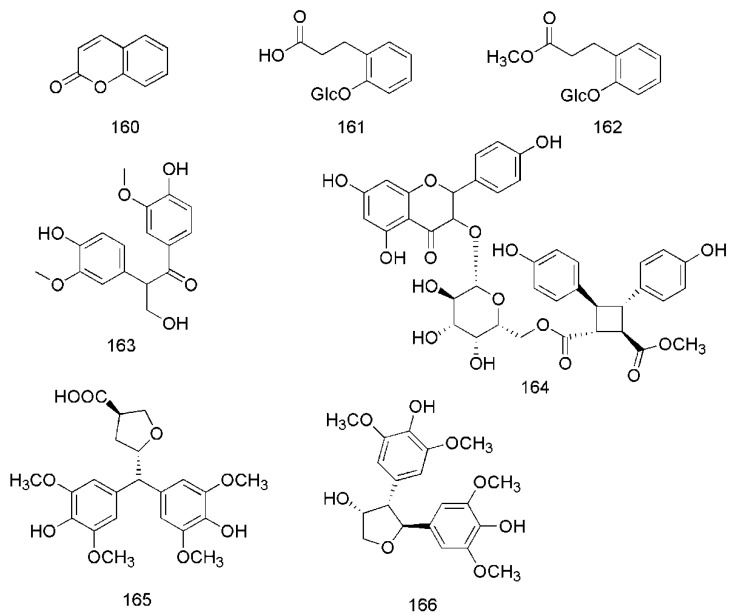
Chemical structures of other compounds found in *C. cassia*.

There have been a lot of studies about the phytochemistry of *C. cassia*, and more than 160 components have been separated and identified from the plant. Among them, terpenoids are the most abundant phytochemicals in *C. cassia*, and phenylpropanoids are the bioactive components, among which cinnamaldehyde is considered as the representative component of this plant, and the indicator component stipulated in the CH.P. In addtion to the chemical components found in the bark, the chemical components of other parts of *C. cassia*, including leaves and twigs, were also reported. The identified compounds are listed in this section and the corresponding structures are also comprehensively presented (Table 2, Figure 2, Figure 3, Figure 4, Figure 5, Figure 6, Figure 7, Figure 8 and Figure 9).

### 3.1. Terpenoids

Terpenoids are the main compounds in essential oil of *C. cassia* (EOC). Plant essential oils have a lot of important biological functions and physiological activities. Essential oils with strong antibacterial, antiviral, antitumor and anti-inflammatory effects are the main characteristic components of the Lauraceae [52,53,54]. The terpenoids in EOC are monoterpenes, diterpenes and sesquiterpenes.

So far, 12 monoterpenes have been found in C. cassia. Among them, *endo*-borneol (**1**) and (−)-α-terpineol (**2**) were isolated from essential oil of twigs of *C. cassia* (EOTC) [33]. Moreover, 1-terpineol (**3**) and *cis*-β-terpineol (**4**) were isolated from essential oil of leaves of *C. cassia* (EOLC) [34]. Other compounds also isolated and identified from the bark, leaves and twigs of *C. cassia*, include α-terpineol (**5**) [34,35], β-bisabolene (**6**), α-bisabolol (**7**) [33,36], linalool (**8**), camphene (**9**), β-pinene (**10**), camphor (**11**) and geranyl acetate (**12**) [36], The corresponding structures of these essential oil components isolated from *C. cassia* are shown in Figure 2.

Diterpenoids are also important active constituents found in *C. cassia*. To date, 25 diterpenoids have been reported in this plant. These compounds are potentially effective natural immunomodulators in the treatment of autoimmune diseases, tumorigenesis, and chronic inflammatory diseases [39]. The diterpenoids isolated from the barks of *C. cassia* include cinnzeylanol (**13**), anhydrocinnzeylanol (**14**), cinnzeylanone (**15**) [37], 2,3-dehydroanhydrocinnzeylanine (**16**), 1-acetylcinncassiol A (**17**), anhydrocinnzeylanine (**18**), 18*S*-cinncassiol A 19-*O*-β-d-glucopyranoside (**19**), 18*R*-cinncassiol A 19-*O*-β-d-glucopyranoside (**20**), 18-hydroxycinnzeylanine (**21**), cinncassiol A (**22**), cinncassiol B (**23**), cinncassiol C (**24**), cinncassiol D (**25**), cinncassiol E (**26**) [38], cinncassiol F (**27**), cinncassiol G (**28**), 16-*O*-β-d-glucopyranosyl-19-deoxycinncassiol G (**29**), cinnacasol (**30**), perseanol (**31**), cinncassiol D_1_ (**32**), D_1_ glucoside (**33**), D_2_ glucoside (**34**), D_3_ glucoside (**35**), D_4_ glucoside (**36**), 18-hydroxyperseanol (**37**) [39]. The corresponding structures of these essential oil components isolated from *C. cassia* are shown in Figure 3.

Sesquiterpenoids are another class of bioactive constituents found in *C. cassia.* Twenty seven sesquiterpenoids, including curcumene (**38**), δ-cadinene (**39**), espatulenol (**40**), caryophyllene oxide (**41**) [33], *trans*-caryophyllene (**42**), germacrene D (**43**) [40], caryophyllene (**44**) [34,35], α-cubebene (**45**), (−)-isoledene (**46**), α-bulnesene (**47**), patchouli alcohol (**48**), α-copaene (**49**) [35], α-muurolene (**50**), α-cadinol (**51**) [33,35], copaene (**52**), isoledene (**53**), 1-(1,5-dimethyl-4-hexenyl)-4-methylbenzene (**54**), cedrene (**55**), α-calacorene (**56**) [36], cinnamoid A (**57**), cinnamoid B (**58**), cinnamoid C (**59**), cinnamoid D (**60**), cinnamoid E (**61**), (−)-15-hydroxy-tmuurolol (**62**), 15-hydroxy-α-cadinol (**63**) and *ent*-4β,10α-dihydroxyaromadendrane (**64**) are reported from this plant [37]. The corresponding structures of these sesquiterpenoids are shown in Figure 4.

### 3.2. Phenylpropanoids

Phenylpropanoids are the main bioactive components of *C. cassia*. In 2013, cinnamaldehyde (**65**) and *cis*-2-methoxycinnamic acid (**66**) were isolated from essential oil of bark of *C. cassia* (EOBC), their contents being 42.37% and 43.06%, respectively [40]. In addition, coniferaldehyde (**63**) [33], *o*-methoxycinnamaldehyde (**68**) [40], 2-methoxycinnamaldehyde (**69**), 2′-methoxycinnamaldehyde (**70**) [33,35], cinnamylalcohol (**71**) [33,36], *cis*-cinnamaldehyde (**72**), *trans*-cinnamaldehyde (**73**), ethyl cinnamate (**74**) [36], eugenol (**75**), cinnamyl acetate (**76**) [34,36], 2-hydroxycinnamic acid (**77**), 2-hydroxycinnamaldehyde (**78**), 4-methoxycinnamaldehyde (**79**), and cinnamic acid (**80**) [41,42] were isolated from different parts of *C. cassia*. The corresponding structures of these phenylpropanoids are shown in Figure 5.

### 3.3. Glycosides

So far, three glycosides have been isolated from the twigs of *C. cassia*, including cinnacasolide A (**81**), cinnacasolide B (**82**) and cinnacasolide C (**83**) [42], two glycosides isolated from the twigs and leaves of *C. cassia* include cinnacasside A (**84**) and cinnacasside C (**85**) [43,44].

Furthermore, 19 glycosides had been isolated from the barks of *C. cassia*, including cinnacasside B (**86**), cinnacasside F (**87**), cinnacasside G (**88**) [45], cinnacassoside D (**89**) [46], cinnacassoside A (**90**), cinnacassoside B (**91**), cinnacassoside C (**92**), 3,4,5-trimethoxyphenol-β-d–apiofuranosyl (1→6)-β-d-glucopyranoside (**93**), 3-trimethoxy-4-hydroxyphenol-β-D–apiofuranosyl(1→6)-β-d-glucopyranoside (**94**), 3,4-dimethoxy-phenol-β-d–apiofuranosyl (1→6)-β-d-glucopyranoside (**95**), (−)-lyoniresinol 3α-*O*-β-d-gluco- pyranoside (**96**) [47], methyl 2-phenylpropanoate-2-*O*-β-d-apiofuranosyl-(1→6)-*O*-β-d–gluco-pyranoside (**97**), cinnacasolide E (**98**), 3,4,5-trimethoxyphenol-β-d-apiofuranosyl-(1→6)-*O*-β-d-glucopyranoside (**99**), samwiside (**100**), phenol-β-d-apiofuranosyl-(1→6)-*O*-β-d-glucopyranoside (**101**), (6*R*,7*R*,8*R*)-7a-[(β-d-glucopyranosyl)oxy]lyoniresinol (**102**), (6*S*,7*R*,8*R*)-7a-[(β-d-gluco-pyranosyl)oxy]lyoniresinol (**103**), (6*R*,7*S*,8*S)*-7a-[(β-d-glucopyranosyl)- oxy]lyoniresinol (**104**) [48]. The corresponding structures of these glycosides isolated from *C. cassia* are shown in Figure 6.

### 3.4. Lignans

In recent years, lignanoids have been found in *C. cassia*, and 26 lignanoids have been isolated from it, which are shown in Figure 7. In 2016, cinncassin E (**105**) was found from the bark of *C. cassia* and its nitric oxide inhibitory activity has been demonstrated. Meanwhile, the lignanoids isolated from the twigs of *C. cassia* include cinncassin D (**106**), picrasmalignan A (**107**), (+)-leptolepisol C (**108**), (−)-(7*R*,8*S*,7′*R*,8′*S*)-syringaresinol (**109**), (+)-isolariciresinol (**110**), (−)-secroisolariciresinol (**111**), (+)-*erythro*-(7*R*,8*S*)-guaiacylglycerol-8-vanillin ether (**112**), (+)-*threo*-(7*S*,8*S*)-guaiacylglycerol-β-coniferyl aldehyde ether (**113**), (+)-*erythro*-(7*S*,8*R*)-guaiacylglycerol-β-coniferyl aldehyde ether (**114**), (−)-*erythro*-(7*R*,8*S*)-guaiacylglycerol-β*-O*-4′-sinapoyl ether (**115**), (−)-*erythro*-(7*S*,8*R*)-syringylglycerol-8-*O*-4′-(sinapoyl alcohol) ether (**116**), (7*S*,8*R*)-lawsonicin (**117**), 5′-methoxylariciresinol (**118**), (+)-(7′*R*,8*R*,8′*R*)-5,5′-dimethoxylariciresinol (**119**), (+)-(7′*S*,8*R*,8′*R*)-5,5′-dimethoxylariciresinol (**120**) [46]. The lignanoids isolated from the leaves of *C. cassia* include cinnacassin F (121), cinnacassin G (122), cinnacassin H (123), cinnacassin I (**124**), cinnacassin J (**125**), cinnacassin K (**126**), cinnacassin L (**127**), cinnacassin M (**128**), cinnacassin N (**129**) and cinnacassin O (**130**) [44].

### 3.5. Lactones

In 2017, cinnamomulactone (**131**), isolated from the twigs of *C. cassia*, was found to have an effect against matrix metalloproteinase (MMP) [49]. Later, 5*R*-methyl-3-heptatriacontyl-2(5*H*)-furanone (**132**) [50], cinncassin A_2_ (**133**), cinncassin A_3_ (**134**), cinncassin A_4_ (**135**), cinncassin A_5_ (**136**), cinncassin A_6_ (**137**), cinncassin A_7_ (**138**) and cinncassin A (**139**) were found from the twigs of *C. cassia* [44]. The corresponding structures of these lactones isolated from *C. cassia* are shown in Figure 8.

### 3.6. Other Compounds

In addition to these major compounds mentioned above, some other chemical compounds are found from *C. cassia*, including benzyl benzoate (**140**), 2-hydroxybenzaldehyde (**141**), 3-phenylpropanol (**142**) [33], 2,2,4,6,6-pentamethylheptane (**143**), 2,5,9-trimethyldecane (**144**), 2-ethyl-5-propylphenol (**145**), 3,4-dimethoxyphenethyl alcohol (**146**), 2,5-dimethylundecane (**147**) [40], benzaldehyde (**148**), phenylethyl alcohol (**149**) [33,40], 2-methoxyphenylacetone (**150**) [35], benzenepropanal (**151**) [33,35], acetophenone (**152**) [33,36], 1,3-dimethylbenzene (**153**), styrene (**154**), 2,2,4-trimethyl-1,3-pentanediol (**155**), decanal (**156**), 2,6,10-trimethyldodecane (**157**) [36], epicatechin-(4β→8)-epicatechin-(4β→8)-epicatechin (**158**) [51], rosavin (**159**) [42,47], coumarin (**160**) [36,49], dihydromelilotoside (**161**), methyl dihydromelilotoside (**162**) [42], evofolin B (**163**) [44,47], cinnamomoside A (**164**) [44], cinncassin C (**165**) and cinncassin B (**166**) [46] (Figure 9).

## 4. Pharmacology

### 4.1. Anti-Tumor Effects

Histone deacetylases (HDACs) are enzymes which play a special role in tissue development and homeostasis. HDACs are divided into four categories: Class I (HDAC1, 2, 3 and 8); II (HDAC4, 5, 6, 7, 9 and 10); and IV (HDAC11) [55]. Recent studies have shown that HDACs are associated with tumor, cardiovascular, autoimmune and neurodegenerative diseases, and HDAC8 plays an important role in the physiological process of these diseases [56]. Gene knockout of HDAC8 can change the growth of cancer cells, cause cell cycle arrest and differentiation of neuroblastoma cells [57]. Trichostatin A (TSA) is a famous antitumor drug and HDAC inhibitor. In 2017, it was found that the bioactive compounds of water extracts of *C. cassia* (WEC, including cinnamic acid, cinnamaldehyde, and cinnamyl alcohol) bind to the active sites of the HDAC8 enzyme like TSA, and the molecular descriptors of *C. cassia* compounds and the binding interactions and energies were similar to those of TSA. Moreover the bioactive components of *C. cassia* were easier to synthesize. These studies showed that *C. cassia* is a potential antitumor drug [58]. *C. cassia* components have been extensively studied in lung cancer, breast cancer, oral cancer, cervical cancer, head and neck squamous cell carcinoma.

Ohnuma et al. studied the inhibitory effect of procyanidins (0–300 μg/mL), bioactive components of *C. cassia*, against the lung cancer cell lines A549, LK-2, abd LU-99 and the potential mechanisms. It found that procyanidins could activate insulin-like growth factor-1 receptor (IGF-1R) phosphorylation and cysteine protease, inhibit the activity of Nrf2-regulated enzymes and the level of Nrf2 expression in lung cancer cells [59,60,61].

In 2013, Kin et al. found that procyanidin C1, isolated from the bark of *C. cassia*, could inhibit TGF- β-induced epithelial-to-mesenchymal transition (EMT) and cell metastasis in A549 lung cancer cells in a dose-dependent manner [51]. Later, in 2017, it was reported that ethanol extracts of *C. cassia* (EEC) possess antimetastatic activity against A549 and H1299 cells by inhibiting TGF-b1-induced EMT and suppressing A549 tumor growth in vivo [62]. In 2018, Wu et al. reported that EEC can inhibit the metastasis of A549 and H1299 tumor cells by repressing u-PA/MMP-2 via FAK to ERK1/2 pathways, and there was no cytotoxicity at the highest concentration of 60 μg/mL [63]. Furthermore, Lee et al. found that water extracts of twigs of *C. cassia* (WETC) could inhibit the growth of the lung cancer cell lines A549, H1299 and LLC by inhibiting the activity of pyruvate dehydrogenase kinase (PDHK) [64].

In 2016, Chang et al. reported that EOC and cinnamaldehyde could significantly suppress the activity of HSC 3 cells and promote their apoptosis, with half maximal inhibitory concentration (IC_50_) values of 13.7 and 10 μg/mL [65]. Later, in 2018, it was reported that ethanol extracts of twigs of *C. cassia* (EETC) can induce oral cancer cell death and inhibit nude mice tumor growth by activating caspase-3 and reducing Bcl-2 to induce apoptosis [66].

In 2015, Sima et al. demonstrated that hexane extracts of barks of *C. cassia* (HEBC) can induce apoptosis of MDA-MB-231 and MCF-7 breast cancer cell lines via stimulating the expression of AKT1 in MCF-7 cells and down-regulating the expression in MDA-MB-231 cells. Meanwhile, activation of caspase-8 is reported for the first time that it is the main apoptotic pathway of *C. cassia* in the treatment of breast cancer [67].

Furthermore, the anti-tumor effects of *C. cassia* on cervical cancers have also been reported in recent years. In 2010, Koppikar et al. reported that water extracts of barks of *C. cassia* (WEBC) can change the growth kinetics of a cervical cancer cell line (SIHA) and down-regulate MMP-2, decreasing the cell mobility in a dose-dependent manner, WEBC can induce apoptosis of cervical cancer cells by increasing intracellular calcium signal and loss of mitochondrial membrane potential [68].

Additionally, an investigation in 2015 indicated that EOTC can significantly inhibit the growth of different cell lines (FaDu, Detroit-562, SCC-25) of head and neck squamous cell carcinoma (HNSCC) by inhibiting the active site of EGFR-TK, and also significantly inhibit the tumor growth in a Hep-2 cell xenotransplantation model [53].

### 4.2. Anti-Inflammatory and Analgesic Effects

In 2012, it was reported that cinnamaldehyde (1.25, 2.5 and 5mg/kg) can decrease paw edema after carrageenan injection, and increase the activities of catalase (CAT), superoxide dismutase (SOD), and glutathione peroxidase (GPx) in the paw tissue. Meanwhile, cinnamic aldehyde (6.25–50 μM) significantly inhibited the levels of nitric oxide (NO), tumor necrosis factor (TNF-α), and prostaglandin E2 (PGE2) levels, blocked protein expression of inducible nitric oxide synthase (iNOS), cyclooxygenase-2 (COX-2), nuclear transcription factor kappa B (NF-κB), and IκBα in Lipopolysaccharide (LPS)-stimulated mouse macrophage (RAW264.7) [69]. In addition, Joung et al. reported that WEBC (20, 100 and 500 mg/kg) can significantly decrease the serum levels of LPS-induced TNF-α and interleukin (IL)-6, and WEBC (10–400 μg/mL) can inhibit inflammatory responses in LPS-stimulated mouse peritoneal macrophages via inhibiting the mRNA expression of TNF-α and the activation of JNK, p38 and ERK1/2 [4].

In 2014, Pannee et al. reported that EOLC and cinnamaldehyde (1.25–20 μg/mL) can decrease the production of NO, the levels of monocyte chemoattractant protein-1 (MCP-1), macrophage inflammatory protein-1α (MIP-1α), TNF-α, IL-1β and IL-6, inhibit the expression of COX-2, iNOS and microsomal prostaglandin-E synthase-1 (mPGES-1) in LPS-activated J774A.1 cells (IC_50_ = 6.1 ± 0.25 and 9.97 ± 0.35 μg/mL, respectively) [34]. A 2015report showed that ethyl acetate extracts of barks of *C. cassia* (EAEBC) can suppress inflammatory responses via the inhibition of NO and TNF-α in LPS-induced RAW 264.7 and J774A.1 macrophages (IC_50_ = 19.7 ± 6.0 μg/mL and 78.4 ± 1.5 μg/mL, LC_50_ = 140 ± 9.0 μg/mL) [70].

In 2016, Lan et al. demonstrated that EOTC (15, 30 and 60 mg/kg) can significantly reduce the amount of writhing induced by oxytocin and acetic acid, inhibit the Complete Freund’s adjuvant (CFA) and formalin-induced paw flinching and licking, in addition, EOTC also inhibited carrageenan-induced mechanical hyperalgesia and paw edema via inhibiting the levels of TNF-α, IL-1β, NO and PGE2, and depressed the expressions of iNOS and COX-2 in paw skin tissue of mice [52]. In addition, EAEBC was reported to be inhibitory to the production of NO in LPS-induced BV-2 microglial cells [46].

In 2017, Shin et al. reported that ethanol extracts of barks of *C. cassia* (EEBC) (25, 50 and 100 mg/kg) can improved the survival rate in the LPS-induced septic shock and gout murine model via inhibiting inflammasome activation including NOD-like receptor 3 (NLRP 3), NLRC4 and interferon-inducible protein AIM2 [71]. Later, cinnamomulactone (0.1, 1, 10 and 100 μM), a new phytocompound from the EETC, was reported to be inhibitory to the expression of MMP-1, MMP-3 and IL-1β in rheumatoid arthritis synovial fibroblasts [49]. Moreover, in 2018, Sharma et al. reported that WEBC (50, 100 and 200 mg/kg) can significantly reduce IL-1, MDA, TNF-α levels and joint swelling in a concentration-dependent manner in rats with CFA-induced and formaldehyde- induced arthritis [72].

Additionally, *C. cassia* was confirmed to inhibit some other kinds of painful diseases. Oxaliplatin, a chemotherapeutic drug, can induce cold and mechanical hypersensitivity, but there is still a lack of effective treatments for neuropathic pain without side effects, it has been found that *C. cassia* has an effective analgesic effect on neuropathic pain induced by oxaliplatin [73]. In 2016, Kim et al. reported that WEBC (100, 200 and 400 mg/kg) have a potent anti-allodynic effect via inhibiting the activation of astrocytes and microglia and decreasing the expression of IL-1β and TNF in the spinal cord after injection with oxaliplatin [74]. Later, In 2019, cinnamic acid (10, 20 and 40 mg/kg), a major compound of *C. cassia*, was reported to provide relief against oxaliplatin-induced neuropathic pain through inhibiting spinal pain transmission [75].

### 4.3. Anti-diabetic and Anti-obesity Effects

In 2006, Kwon et al. found that the WEBC (100, 250 and 500 mg/kg) can completely prevent streptozotocin (STZ)-induced diabetes in mice via inhibiting the expression of iNOS and the activation of NF-κB, Moreover, WEBC (0.125, 0.25, 0.5 and 1.0 mg/mL) decreased the production NO and the expression of iNOS mRNA induced by IL-1 β and TNF-γ, which can completely protect rat insulinoma RINm5F cells against IL-1 β and TNF- γ-induced cytotoxicity [3]. In 2013, Jang et al. found that the polyphenols of *C. cassia* (10 and 50 mg/kg) exhibited strong hypoglycemic activity in STZ-induced diabetes mice [76]. A report in 2014 demonstrated that acetone extract of barks of *C. cassia* (AEBC) showed great potential of decreasing the plasma glucose level via inhibiting rat α-glucosidase, maltase and sucrase activity (IC_50_ = 0.474, 0.38 and 0.10 mg/mL) [77]. Later, Krishna et al. reported that decoumarinated extracts of *C. cassia* (200 mg/kg) can significantly alter the level of blood glucose, serum insulin, lipid distribution and liver antioxidant enzymes in STZ induced diabetic rats [78].

In addition to its hypoglycemic effect, cinnamon can also alleviate some complications of diabetes. In 2013, Luo et al. found that EEBC (10 μM) resisted the growth of high-glucose-induced mesangial cells via depressing the expression of IL-6, collagen IV and fibronectin [47]. Moreover, Yan et al. revealed that EEBC (10, 30 and 50 μg/mL) restrained the expression of fibronectin, MCP-1 and IL-6 in high- glucose-stimulated mesangial cells [37]. In 2018, the extracts of barks of *C. cassia* (EBC) was reported to reduce the levels of MDA and NO, increase glutathione peroxidase (GPx) and glutathione (GSH), and down-regulate iNOS in thoracic aorta to prevent chronic complications of experimentally induced type II diabetes [79]. Furthermore, *C. cassia* silver nanoparticles (CcAgNPS) (5, 10 and 200 mg/kg) showed remarkable mitigation of severe distortion of the glomerular network, had a regenerative potential in diabetes-induced kidney damage [80].

In 2016, Lee et al. reported that the extracts of *C. cassia* (EC) (50, 100 and 200 μg/mL) boosted lipid storage in white adipocytes and increase the fatty acid oxidation capacity throughout the initiation stage of differentiation, which can prevent obesity-induced type II diabetes [81]. In vivo, WEBC (100, 300 mg/kg) significantly decreased serum levels of glucose, insulin, total cholesterol and ALT, suppressed lipid accumulation in liver, prevented oral glucose tolerance and insulin resistance in obese mice. In vitro, WEBC (0.1 and 0.2 mg/mL) increased ATP levels by increasing the mRNA expressions of mitochondrial biogenesis-related factors in C2C12 myoblast [82].

### 4.4. Antibacterial and Antiviral Effects

The abuse of antibiotics has led to the emergence of drug-resistant bacteria. Plant essential oils have a wide range of bacteriostatic effects and are rarely suffer from resistance issues. In 2013, Zhao et al. reported that EOBC had notable potent activities against *Staphylococcus aureus, Aspergillus niger, Bacillus subtilis,* and *Escherichia coli* with minimum inhibitory concentration (MIC) values of 200, 200, 200 and 100 μL/mL, respectively [54]. Later, a study revealed that EOBC exhibited strong activity against *Staphylococcus aureus, Escherichia coli, Klebsiella pneumoniae* and *Pseudomonas aeruginosa* with MIC values of 0.28, 0.28, 0.56 and 1.11 mg/mL, respectively [83]. In 2014, Sheng et al. found that EOBC significantly inhibited growth of non-O157 STECs (including O26, O45, O103, O111) with MIC values of 0.025% (*v*/*v*) [84]. By using the agar diffusion method, the antibacterial activity of EOBC on *Escherichia coli* and *Staphylococcus* was evaluated, and the MIC values were both 1.0 mg/mL [85]. In 2018, Katy et al. reported that EOC inhibited both *Staphylococcus hyicus* and *Staphylococcus aureus* with MIC values of 0.078% [86]. Moreover, Li et al. revealed that *Propionibacterium acnes, Staphylococcus epidermidis* and *Staphylococcus aureus* were very sensitive to EOBC, and the MIC values were 0.156, 0.313 and 0.25 μL/mL [87]. In vitro, the EOC exhibited strong inhibition against *Staphylococcus aureus* with MIC values of 500 μL/L, and it had no inductive effect on the acquisition of stress tolerance in *S. aureus* [88]. Using the agar disc diffusion assay, the antibacterial activity of EOBC on *Escherichia coli, Staphylococcus aureus,* and *Pseudomonas aeruginosa* was evaluated, and the MIC values were 4.88, 4.88, and 19.53 μg/mL [89].

Molecular diversity in plants has helped humans discover many effective drugs, such as quinine in *Cinchona succirubra* and artemisinin in *Artemisia annua*, leading to a shift in anti-malaria research focus. In 2016, CcAgNPS was demonstrated that inhibited H7N3 influenza A virus in Vero cells with MIC values of 125 μg/mL, and it was found that had no-toxicity to Vero cells at the concentration of 500 μg/mL [90].

### 4.5. Cardiovascular Protective Effects

In 2015, Kwon et al. found that WEBC (10, 30 and 50 μg/mL) inhibited the proliferation of VSMCs via arresting G_0_/G_1_ and down-regulating the expression of cell cycle positive regulatory proteins (p21 and p27), which can improve cardiovascular disease caused by proliferation of vascular smooth muscle cells [91]. Later, WEC (0–100 μg/mL) was demonstrated to inhibit the phosphorylation of ERK, p38 and vascular endothelial growth factor (VEGF) R2, the activation of MMP and VEGF-induced proliferation, migration, invasion, tube formation in cultured human umbilical vein endothelial cells (HUVECs) [92]. Furthermore, in vivo, Wei et al. revealed that WEBC (750 mg/kg) significantly decreased the serum levels of TG, TC, LDL and BNP shortened the intervals of QRS and P-R, increased the Ca^2+^Mg^2+^-ATP enzyme activity and the contents of PCr, ATP and ADP in STZ-induced myocardial injury diabetic rats [93].

### 4.6. Cytoprotective Effects

In 2013, it was reported that *C. cassia* powder (CP) (2, 10 and 100 mg/g per feed) protected against gastric ulcers induced by stress, ethanol or HCl through a cytoprotective mechanism [94]. Later, In 2016, El-Kady et al. found that WEBC (10–50 μg/mL) resisted the cytotoxic effect of *cis*-diammine dichloroplatinum (CDDP) in vitro via preventing CDDP-induced increased expression of mitochondrial Bax protein, releasing of mitochondrial cytochrome c, caspase-3 activation, DNA fragmentation and generation of ROS, up-regulating expression of the cytoprotective gene (heme oxygenase (HO)-1) [95].

### 4.7. Neuroprotective Effects

Recently, investigations into the neuroprotective effects of *C. cassia* such as anti-anxiety, cognitive improvement and anti-depressant have been conducted. In 2007, an experimental study on anti-anxiety effects showed that EEBC significantly increased the percentage of entries into and the time spent in the open arms in the elevated plus maze (EPM) test via regulating the 5-hydroxytryptamine1A (5-HT_1A_) and γ-aminobutyric acid (GABA)-ergic system [96]. Further, Jung et al. reported that the anxiolytic-like effects of EEBC (100, 750 mg/mL) were mediated by region-specific changes of 5-HT_1A_ receptors in the dorsal raphe nucleus [97].

In 2011, the WEC was evaluated for cognitive improvement in vitro and in vivo. The results showed that WEC (1, 10 and 100 μg/mL) markedly inhibited the formation of toxic Ab oligomers and prevented the toxicity of Ab on neuronal PC12 cells with the MIC value of 0.7 μg/mL. In AD fly model, WEC (0.75 mg/mL) extend their life, recovered their locomotion defects and totally eliminated tetrameric species of β-amyloid polypeptide (Aβ) in their brain. Moreover, WEC (100 μg/mL) marked decreased 56 kDa Aβ oligomers, reduced plaques and improved the cognitive functions in AD transgenic mice model [98]. Later, In 2017, it was reported that total flavonoids of Cinnamomi Cortex (20–100 μg/mL) enhanced viability of PC12 cell and activity of SOD, alleviated the DNA damage, decreased the expression levels of the Bax/Bcl-2 rate, cl Caspase-9 and the content of MDA in 6-hydroxydopamine injured PC-12 cell [99]. In 2016, the EC was evaluated for anti-depressant in vivo. The results showed that EC (25 and 50 mg/kg) significantly decreased the immobility time in TST (tail suspension test) and increased the 5-HTP-induced head twitches via rising the levels of serotonin [100].

### 4.8. Immunoregulation Effects

In 2014, Zeng et al. studied the immunoregulation effect of EEBC, the results showed that EEBC (100 μg/mL) inhibited 78.5% of T cell proliferation induced by concanavalin A (ConA), moreover, cinncassiol G and cinnacasol (50 and 100 μM), two new phytocompounds from Cinnamomi Cortex, significantly inhibited T cell proliferation induced by ConA and B cell proliferation induced by LPS in a dose dependent manner, and had no cytotoxicity in mice lymphocytes [39]. In addition, it is reported that phenolic glycosides of Cinnamomi Cortex (12.5–200 μM) inhibited T cell proliferation induced by ConA [48]. Furthermore, In 2017, cinnacasside F (400 μM), a new glycosides from Cinnamomi Cortex, inhibited 36.1% T cell proliferation and 20.3% B cell proliferation [45].

### 4.9. Anti-Tyrosinase Activity

In 2013, Chang et al. found that EOBC and *trans*-cinnamaldehyde had remarkably anti-tyrosinase activity (IC_50_ = 6.16 ± 0.04 and 4.04 ± 0.08 mg/mL, respectively) [40]. Moreover, Chou et al. reported that EOC (1.0, 2.0, 2.5 and 5.0 μg/mL) and *trans*-cinnamaldehyde (1.0, 2.0 and 2.5 μg/mL) reduced the melanin content and tyrosinase activity of the cells, down-regulated tyrosinase expression without exhibiting cytotoxicity, decreased thiobarbituric acid-reactive substance (TBARS) levels and restored glutathione (GSH) and catalase activity in murine B16 melanoma cells stimulated with α-melanocyte-stimulating hormone (α-MSH) (IC_50_ = 6.16 ± 0.04 and 4.04 ± 0.08 mg/mL, respectively) [101].

### 4.10. Other Pharmacological Effects

Apart from the pharmacological effects displayed above, *C. cassia* also possesses some other activities. In 2012, cinnamaldehyde, 2-methoxycinnamaldehyde, 2-hydroxycinnamaldehyde, cinnamic acid and coniferaldehyde and *O*-coumaric acid isolated from extracts of twigs of *C. cassia* (ETC) showed significantly inhibitory action on xanthine (IC_50_ = 7.8–36.3 μg/mL) [42]. In 2014, the effect of methanol extracts of *C. cassia* (MEC) on arginase and sexual function were evaluated in vitro and in vivo. The results showed that MEC (0.1, 1, 10, and 100 μg/mL) inhibited arginase activity with an IC_50_ of 61.72 ± 2.20 μg/mL in rat corpus cavernosum smooth muscle (CCSM). In addition, MEC (100 mg/kg) increased smooth muscle level, decreased collagen level in rat penile tissue and increased sexual function of young male rats [102]. Moreover, In 2017, it is reported that methanol extracts of barks of *C. cassia* (MEBC) (50, 100 and 150 mg/kg) aided in the recovery of the antioxidant system as well as protective role in histological damages and some haematological parameters in the rat liver treated with titanium dioxide nanoparticles (TiO_2_ NPS) or titanium dioxide bulk salt (TiO_2_ bulk salt) via increasing the serum level of CAT, decreasing the levels of SOD, lipid peroxidation, alanine aminotransaminase (ALT), aspartate aminotransferase (AST) and alkaline phosphatase (ALP)[103]. Later, the effect of EOTC on uterine contraction was evaluated in vitro and in vivo. The results showed that EOTC (25, 50 and 100 μg/mL) inhibited spontaneous uterus contractions in a dose-dependent manner via inhibiting the level of Ca^2+^ in Myometrial cells (IC_50_ = 361.3 μg/mL). In addition, EOTC (15, 30 and 60 mg/kg) reduced oxytocin (OT)-induced writhing responses via decreasing the level of PGF_2α_, COX-2 and phosphorylation of myosin light chain 20 (P-MLC 20) [104].

Visceral leishmaniasis (VL) or kala-azar is the fatal form of leishmaniasis caused by *Leishmania donovani*, outbreaks in the tropics and subtropics, producing physical and reproductive disabilities and causing an immense death toll of 20,000–40,000 each year, which have made a great impact on society [105]. Despite decades of research, there is no available commercial vaccine against VL and chemotherapy is failing owing to emerging resistance and adverse side effects [106]. In 2019, Afrin et al. found that EBC showed great anti-promastigote activity through inducing apoptosis in vitro with IC_50_ values of 33.66 ± 3.25 μg/mL. In addition, EBC (50 and 100 mg/kg) showed significant protection against *L. donovani* infected mice and hamsters, the in vivo protection achieved was 80.91% (liver) and 82.92% (spleen) in mice and 75.61% (liver) and 78.93% (spleen) in hamsters. The results showed that Cinnamomi Cortex had direct antileishmanial activity and non-toxicity in vitro and in vivo [107].

### 4.11. Summary of Pharmacologic Effects

In conclusion, *C. cassia* has a wide range of pharmacological effects including anti-tumor effects, anti-inflammatory and analgesic effects, anti-diabetic and anti-obesity effects, antibacterial and antiviral effects, cardiovascular protective effects, cytoprotective effects, neuroprotective effects, immunoregulation effects and anti-tyrosinase activity (Table 3). Modern pharmaceutical research mainly focuses on extracts and chemical components, which indicated the prospects of *C. cassia* in the treatment of such diseases.

## 5. Toxicity

*C. cassia*, as a common flavor and medicinal material, has little toxicity, and there are few reports about the toxicity and clinical adverse reactions of *C. cassia*. In 2002, it was reported that a 47 year- old male patient had swelling and itching in both hands and face after touching steamed Cinnamomi cortex for 1 h, while the other two people in the same group were normal. After disengagement, 10% calcium gluconate 10 mL and 50% glucose 20 mL was given via intravenous injection for 3 days, and the swelling gradually subsided [108]. In 2005, it is reported for the first time that the use of *C. cassia* essential oil mud bath could cause extensive eczematous and bullous dermatitis [109]. Later, in a randomized controlled trial of *C. cassia* hemoglobin A1C reduction in patients with type 2 diabetes, the treatment group received two *C. cassia* capsules (500 mg each) per day for 90 days, and one of the subjects developed a rash, which subsided after discontinuation, but no further adverse reactions were observed [110]. In 2018, a 13-week repeat-dose oral toxicity study revealed that body weights of rats were normal, the weight of kidney/live and the level of total cholesterol were increased after receiving WEBC at up to 2000 mg/kg, but it was not mutagenic or clastogenic [111]. Later, a 8-week repeat-dose oral toxicity study revealed that renal function showed a significant increase, kidney and liver histology showed distortions in hepatocytes and sinusoidal linings with infiltrations, degenerative changes in glomerular and Bowman’s capsules with fibrillary mesangial interstitium after receiving CcAgNPs at up to 200 mg/kg [112]. In summary, *C. cassia* essential oil may cause skin irritation and its extract may possess potential nephrotoxicity and hepatotoxicity at dose higher than its recommended daily safe dose.

## 6. Conclusions and Future Perspectives

In conclusion, the traditional usages, phytochemistry, pharmacological activity and toxicity of *C. cassia* have been summarized in the present review. Modern studies have confirmed that *C. cassia* has a wide range of pharmacological activities, including anti-tumor effects, anti-inflammatory and analgesic effects, anti-diabetic and anti-obesity effects, antibacterial and antiviral effects, for which it has been used in the clinic in many countries. Moreover, *C. cassia* has the same origin as a medicine and food which is often used as a condiment in our daily life. Nevertheless, there is still a lack of sufficient research about the alimentotherapy, health products, toxicity and side effects of *C. cassia*. Therefore, more investigations need to be done in *C. cassia* in the future.

Firstly, there is a lack of systematic toxicity and side effects studies of the extracts or compounds isolated from *C. cassia*. Essential oils are the main constituents of *C. cassia*, which has been reported to irritate the skin and possibly cause allergies, and the antibacterial effect of essential oil is applied in food and cosmetics. In addition, as a plant with the same origin as medicine and food, people will also eat *C. cassia* for a long time, therefore, in-depth investigations on its toxicity and side effects are a guarantee for the safe use of this plant. Secondly, with the attention humans attach to health preservation, alimentotherapy and health care products are being more and more widely used, thus, there is great space for the development of valuable *C. cassia* health products. Thirdly, for traditional medicinal uses, the bark and twigs of *C. cassia* are important components of traditional Chinese medicine formula, such as Guifu-lizhong pills [113], Guizhi-shaoyao-zhimu decoction [114], thus current pharmacological activity studies of *C. cassia* have mainly focused on its barks and twigs, and there are few investigations on the leaves, fruits and other parts of *C. cassia*. Thus the study on other parts of *C. cassia* may be helpful to the development of alternative medicines and new drugs. Fourthly, the bark is an important part of the tree body, which can maintain the temperature, prevent diseases and pests, its main function is to transport nutrients for the tree body. The barks of *C. cassia* are officially recognized as *Rou-Gui* in the CH.P (2015 Edition), but many other *Cinnamomum* species such as *Cinnamomum zeylanicum* and *Cinnamomum burmannii* Blume, are used as *C. cassia* alternatives in many countries. Therefore, the plant morphology, chemical compositions and pharmacological activities should be used to differentiate the different varieties, and it is important to safeguard the efficacy of *C. cassia* to ensure its suitability and security for clinical use. Fifthly, the *C. cassia* was traditionally used in the treatment of dyspepsia, gastrointestinal diseases, irregular menstruation and arthritis, etc., but not all of these uses had been confirmed by modern preliminary studies. The discovery of artemisinin was based on a classical Chinese medical monograph, therefore the traditional uses in classical monographs should be reasonably developed, and more potential pharmacological activity of *C. cassia* might be explored in the future.

## Figures and Tables

**Table 1:** The traditional and clinical uses of *C. cassia* in China.

Prescription Name	Main Component	Traditional and Clinical Uses	Reference
Zi Shen Pills	Anemarrhenae Rhizoma, Phellodendri Chinensis Cortex, Cinnamomi Cortex	Treating dysuria due to accumulation heat in bladder	[7]
Gui Fu Li Zhong Pills	Cinnamomi Cortex, Aconiti Lateralis Radix Praeparata, Codonopsis Radix, Glycyrrhizae Radix Et Rhizoma, Atractylodis, Macrocephalae Rhizoma, Roasted Ginger	Curing abdominal pain, diarrhoea and vomiting due to deficient cold of spleen and stomach,	[8]
Ding Gui Wen Wei San	Caryophylli Flos, Cinnamomi Cortex	Curing abdominal pain caused by cold syndrome	[9]
Jian Wei Shi Wei Pills	Granati Pericarpium, Amomi Fructus Rotundus, Chebulae Fructus, Cinnamomi Cortex, Piperis Fructus, Kaempferiae Rhizoma, Piperis Longi Fructus	Curing cacochylia, gasteremphraxis, vomiting and diarrhea	[10]
Qi Wei Wei Tong Capsules	Aucklandiae Radix, Piperis Longifructus, Alpiniae Officinaru Mrhizoma, Galli Gigerii Endothelium Corneum, Euodiae Fructus, Cinnamomi Cortex	Treating diarrhoea, vomiting, poor appetite, gastroduodenal ulcer and superficial gastritis	[11]
Qi Wei Pu Tao San	Gypsum Fibrosum, Carthami Flos, Glycyrrhizae Radix Et Rhizoma, Cyperi Rhizoma, Cinnamomi Cortex, Granati Pericarpium	Treating cough, asthma and chest tightness due to overwork and weakness	[12]
Wu Wei Qing Zhuo San	Granati Pericarpium, Carthami Flos, Carthami Flos, Cinnamomi Cortex, Piperis Longi Fructus	Treating poor appetite, dyspepsia, gastralgia, belching, abdominal distention and diarrhea	[13]
Wu Ling San	Poria, Alismatis Rhizoma, Polyporus, Cinnamomi Cortex, Atractylodis Macrocephalae Rhizoma	Treating dysuria, oedema, abdominal distension, diarrhoea and vomiting	[14]
Zhong Jing Wei Ling Pills	Cinnamomi Cortex, Corydalis Rhizoma, Ostreae Concha, Foeniculi Fructus, Amomi Fructus, Alpiniae Officinarum Rhizoma, Paeoniae Radix Alba, Glycyrrhizae Radix Et Rhizoma	Treating poor appetite, stomachache, abdominal distension and diarrhoea due to weak spleen and stomach	[15]
Er Xie Kang Tiemo	Caryophylli Flos, Piperis Fructus, Euodiae Fructus, Cinnamomi Cortex	Curing non-infectious diarrhea in children	[16]
Ba Wei Rou Gui Capsules	Cinnamomi Cortex, Aucklandiae Radix, Paeoniae Radix Alba, Piperis Longi Fructus, Foeniculi Fructus, Amomi Fructus Rotundus, Alpiniae Officinarum Rhizoma, Glycyrrhizae Radix Et Rhizoma	Curing stomachache, poor appetite and dyspepsia due to asthenia cold of spleen and stomach	[17]
Li Er Mian Capsules	Coptidis Rhizoma, Cinnamomi Cortex	Treating cardiopalmus, insomnia and dreamful sleep	[18]
Qian Lie Gui Huang Pills	Rhei Radix Et Rhizoma, Gleditsiae Fructus Abnormalis, Cinnamomi Cortex, Typhae Pollen, Talcum, Cyathulae Radix	Treating hyperplasia of prostate gland	[19]
Shi Quan Da Bu Tang Jiang	Codonopsis Radix, Atractylodis Macrocephalae Rhizoma, Poria, Glycyrrhizae Radix Et Rhizoma, Angelicae Sinensis Radix, Aconiti Radix Cocta, Paeoniae Radix Alba, Rehmanniae Radix Praeparata, Astragali Radix, Cinnamomi Cortex	Treating pallor, dizziness and palpitation, spontaneous perspiration, weariness of body, cold feet due to deficiency of Qi and blood	[20]
Shi Di Shui	Borneolum, Zingiberis Rhizoma, Rhei Radix Et Rhizoma, Foeniculi Fructus, Cinnamomi Cortex, Capsici Fructus	Treating dizziness, nausea, abdominal pain, gastrointestinal discomfort caused by heat stroke.	[21]
Shen Gui Li Zhong Pills	Ginseng Radix Et Rhizoma, Cinnamomi Cortex, Aconiti Lateralis Radix Praeparata, Zingiberis Rhizoma, Atractylodis Macrocephalae Rhizoma, Glycyrrhizae Radix Et Rhizoma	Curing acrohypothermy, gastrofrigid vomiting, hernia, algomenorrhea, abdominal pain and diarrhea due to deficiency of Yang Qi and asthenia cold of spleen and stomach	[22]
Gu Ben Tong Xue Granules	Cynomorii Herba, Cuscutae Semen, Cinnamomi Cortex, Morindae Officinalis Radix, Astragali Radix, Dioscoreae Rhizoma, Aconiti Lateralis Radix Praeparata, Lycii Fructus, Codonopsis Radix, Epimedi Folium	Treating mild primary thrombocytopenic purpura	[23]
Fu Fang Zao Fan Pills	Melanteritum, Panacis, Quinquefolii Radix, Hippocampus, Cinnamomi Cortex, Jujubae Fructus, Juglandis Semen	Treating aplastic anemia, aleucocytosis, thrombocytopenia, myelodysplastic syndrome	[24]
Xiao Er Fu Xie Tie	Caryophylli Flos, Cinnamomi Cortex, Piperis Longi Fructus	Treating non-infectious diarrhea in children duo to asthenia cold of spleen and stomach	[25]
Shao Fu Zhu Yu Granules	Cinnamomi Cortex, Angelicae Sinensis Radix, Typhae Pollen, Paeoniae Radix Rubra, Foeniculi Fructus, Corydalis Rhizoma, Myrrha, Aconiti Radix Cocta	Treating irregular menstruation, dysmenorrhea, lumbago and leukorrhea due to blood stasis and cold	[26]
Xin Bao Pills	Daturae Flos, Ginseng Radix Et Rhizoma, Cinnamomi Cortex, Aconiti Lateralis Radix Praeparata, Borneolum Syntheticum, Notoginseng Radix Et Rhizoma	Treating chronic cardiac insufficiency, bradycardia and angina pectoris	[27]
Xin Tong Ning Di Wan	Cinnamomi Cortex, Aconiti Radix Cocta, Cyperi Rhizoma	Treating coronary disease and angina pectoris	[28]
You Gui Pills	Rehmanniae Radix Praeparata, Aconiti Lateralis Radix Praeparata, Cinnamomi Cortex, Dioscoreae Rhizoma, Corni Fructus, Cuscutae Semen, Lycii Fructus, Angelicae Sinensis Radix, Eucommiae Cortex	Treating listlessness, spermatorrhea, asynodia, loose stool and frequent micturition due to deficiency of Yang Qi of kidney	[29]
Shi Wei Fu Zheng Granules	Ginseng Radix Et Rhizoma, Rehmanniae Radix Praeparata, Atractylodis Macrocephalae Rhizoma, Astragali Radix, Poria, Paeoniae Radix Alba, Angelicae Sinensis Radix, Cinnamomi Cortex, Glycyrrhizae Radix Et Rhizoma, Aconiti Radix Cocta	Treating aleucocytosis, decrease of immune function caused by tumor radiotherapy and chemotherapy	[30]
Ba Wei Shen Qi Pills	Rehmanniae Radix Praeparata, Dioscoreae Rhizoma, Poria, Schisandrae Chinensis Fructus, Cinnamomi Cortex, Alismatis Rhizoma, Aconiti Lateralis Radix Praeparata, Moutan Cortex	Treating edematous, cough, dyspnea, frequent micturition and loose stool duo to deficiency Yang of kidney	[31]
Dai Wen Jiu Gao	Capsici Fructus, Cinnamomi Cortex, Zineiberis Rhizoma Rrcens, Cinnamon Oil	Curing chronic rheumatic arthritis, chronic gastroenteritis	[32]

**Table 2:** Chemical constituents isolated from *C. cassia*.

Classification	No.	Chemical Component	Part of Plant	Ref.
Terpenoids	1	*endo*-borneol	Twig	[33]
	2	(−)-α-terpineol	Twig	[33]
	3	1-terpineol	Leaves	[34]
	4	*cis*-β-terpineol	Leaves	[34]
	5	α-terpineol	Bark, leaves	[34,35]
	6	β-bisabolene	Bark, twig	[33,36]
	7	α-bisabolol	Bark, twig	[33,36]
	8	linalool	Bark	[36]
	9	camphene	Bark	[36]
	10	β-pinene	Bark	[36]
	11	camphor	Bark	[36]
	12	geranyl acetate	Bark	[36]
	13	cinnzeylanol	Bark	[37]
	14	anhydrocinnzeylanol	Bark	[37]
	15	cinnzeylanone	Bark	[37]
	16	2,3-dehydroanhydrocinnzeylanine	Bark	[38]
	17	1-acetylcinncassiol A	Bark	[38]
	18	anhydrocinnzeylanine	Bark	[38]
	19	18*S*-cinncassiol A 19-*O*-β-d-glucopyranoside	Bark	[38]
	20	18*R*-cinncassiol A 19-*O*-β-d-glucopyranoside	Bark	[38]
	21	18-hydroxycinnzeylanine	Bark	[38]
	22	cinncassiol A	Bark	[38]
	23	cinncassiol B	Bark	[38]
	24	cinncassiol C	Bark	[38]
	25	cinncassiol D	Bark	[38]
	26	cinncassiol E	Bark	[38]
	27	cinncassiol F	Bark	[39]
	28	cinncassiol G	Bark	[39]
	29	16-*O*-β-d-glucopyranosyl-19-deoxycinncassiol G	Bark	[39]
	30	cinnacasol	Bark	[39]
	31	perseanol	Bark	[39]
	32	cinncassiol D_1_	Bark	[39]
	33	D_1_ glucoside	Bark	[39]
	34	D_2_ glucoside	Bark	[39]
	35	D_3_ glucoside	Bark	[39]
	36	D_4_ glucoside	Bark	[39]
	37	18-hydroxyperseanol	Bark	[39]
	38	curcumene	Twig	[33]
	39	δ-cadinene	Twig	[33]
	40	espatulenol	Twig	[33]
	41	caryophyllene oxide	Twig	[33]
	42	*trans*-caryophyllene	Bark	[40]
	43	germacrene D	Bark	[40]
	44	caryophyllene	Bark, leaves	[34,35]
	45	α-cubebene	Bark	[35]
	46	(–)-isoledene	Bark	[35]
	47	α-bulnesene	Bark	[35]
	48	patchouli alcohol	Bark	[35]
	49	α-copaene	Bark	[35]
	50	α-muurolene	Bark, twig	[33,35]
	51	α-cadinol	Bark, twig	[33,35]
	52	copaene	Bark	[36]
	53	isoledene	Bark	[36]
	54	1-(1,5-dimethyl-4-hexenyl)-4-methylbenzene	Bark	[36]
	55	cedrene	Bark	[36]
	56	α-calacorene	Bark	[36]
	57	cinnamoid A	Bark	[37]
	58	cinnamoid B	Bark	[37]
	59	cinnamoid C	Bark	[37]
	60	cinnamoid D	Bark	[37]
	61	cinnamoid E	Bark	[37]
	62	(−)-15-hydroxytmuurolol	Bark	[37]
	63	15-hydroxy-α-cadinol	Bark	[37]
	64	*ent*-4β,10α- dihydroxyaromadendrane	Bark	[37]
Phenylpropanoids	65	cinnamaldehyde	Bark	[40]
	66	*cis*-2-methoxycinnamic acid	Bark, twig, leaves	[40]
	67	coniferaldehyde	Twig	[33]
	68	*o*-methoxycinnamaldehyde	Bark	[40]
	69	2-methoxycinnamaldehyde	Bark, twig	[33,35]
	70	2′-methoxycinnamaldehyde	Bark, twig	[33,35]
	71	cinnamylalcohol	Bark, twig	[33,36]
	72	*cis*-cinnamaldehyde	Bark	[36]
	73	*trans*-cinnamaldehyde	Bark	[36]
	74	ethyl cinnamate	Bark	[36]
	75	eugenol	Bark, leaves	[34,36]
	76	cinnamyl acetate	Bark, leaves	[34,36]
	77	2-hydroxycinnamic acid	Bark, twig	[41,42]
	78	2-hydroxycinnamaldehyde	Bark, twig	[41,42]
	79	4-methoxycinnamaldehyde	Bark, twig	[41,42]
	80	cinnamic acid	Bark, twig	[41,42]
Glycosides	81	cinnacasolide A	Twig	[42]
	82	cinnacasolide B	Twig	[42]
	83	cinnacasolide C	Twig	[42]
	84	cinnacasside A	Bark, twig	[43,44]
	85	cinnacasside C	Bark, twig	[43,44]
	86	cinnacasside B	Bark	[45]
	87	cinnacasside F	Bark	[45]
	88	cinnacasside G	Bark	[45]
	89	cinnacassoside D	Bark	[46]
	90	cinnacassoside A	Bark	[47]
	91	cinnacassoside B	Bark	[47]
	92	cinnacassoside C	Bark	[47]
	93	3,4,5-trimethoxyphenol-β-d–apiofuranosyl (1→6)-β-d-glucopyranoside	Bark	[47]
	94	3-trimethoxy-4- hydroxyphenoll-β-d–apiofuranosyl (1→6)-β-d-glucopyranoside	Bark	[47]
	95	3,4-dimethoxyphenol-β-d–apiofuranosyl (1→6)-β-d-glucopyranoside	Bark	[47]
	96	(−)-lyoniresinol 3α-*O*-β-d-glucopyranoside	Bark	[47]
	97	methyl-2-phenylpropanoate-2-*O*-β-dapiofuranosyl-(1→6)-*O*-β-d–glucopyranoside	Bark	[48]
	98	cinnacasolide E	Bark	[48]
	99	3,4,5-trimethoxyphenol-β-d-apiofuranosyl-(1→6)-*O*-β-d-glucopyranoside	Bark	[48]
	100	Samwiside	Bark	[48]
	101	phenol-β-d-apiofuranosyl-(1→6)-*O*-β-d-glucopyranoside	Bark	[48]
	102	(6*R*,7*R*,8*R*)-7a-[(β-d-glucopyranosyl) oxy] lyoniresinol	Bark	[48]
	103	(6*S*,7*R*,8*R*)-7a-[(β-d-glucopryanosyl) oxy] lyoniresinol	Bark	[48]
	104	(6*R*,7*S*,8*S*)-7a-[(β-d-glucopyranosyl) oxy] lyoniresinol	Bark	[48]
Lignans	105	cinncassin E	Bark	[46]
	106	cinncassin D	Bark	[46]
	107	picrasmalignan A	Bark	[46]
	108	(+)-leptolepisol C	Bark	[46]
	109	(−)-(7*R*,8*S*,7′*R*,8′*S)*-syringaresinol	Bark	[46]
	110	(+)-isolariciresinol	Bark	[46]
	111	(−)-secroisolariciresinol	Bark	[46]
	112	(+)-erythro-(7*R*,8*S*)-guaiacylglycerol-8-vanillin ether	Bark	[46]
	113	(+)-threo-(7*S*,8*S*)-guaiacylglycerol-β-coniferyl aldehyde ether	Bark	[46]
	114	(+)-erythro-(7*S*,8*R*)-guaiacylglycerol-β-coniferyl aldehyde ether	Bark	[46]
	115	(−)-erythro-(7*R*,8*S*)-guaiacylglycerol-β-*O*-4′-sinapoyl ether	Bark	[46]
	116	(−)-erythro-(7*S*,8*R*)-syringylglycerol-8-*O*-4′- (sinapoyl alcohol) ether	Bark	[46]
	117	(7*S*,8*R*)-lawsonicin	Bark	[46]
	118	5′-methoxylariciresinol	Bark	[46]
	119	(+)-(7′*R*,8*R*,8′*R*)-5,5′-dimethoxylariciresinol	Bark	[46]
	120	(+)-(7′*S*,8*R*,8′*R*)-5,5′-dimethoxylariciresinol	Bark	[46]
	121	cinnacassin F	Twig	[44]
	122	cinnacassin G	Twig	[44]
	123	cinnacassin H	Twig	[44]
	124	cinnacassin I	Twig	[44]
	125	cinnacassin J	Twig	[44]
	126	cinnacassin K	Twig	[44]
	127	cinnacassin L	Twig	[44]
	128	cinnacassin M	Twig	[44]
	129	cinnacassin N	Twig	[44]
	130	cinnacassin O	Twig	[44]
Lactones	131	cinnamomulactone	Twig	[49]
	132	5*R*-methyl-3-heptatriacontyl-2(5H)-furanone	Twig	[50]
	133	cinncassin A_2_	Twig	[44]
	134	cinncassin A_3_	Twig	[44]
	135	cinncassin A_4_	Twig	[44]
	136	cinncassin A_5_	Twig	[44]
	137	cinncassin A_6_	Twig	[44]
	138	cinncassin A_7_	Twig	[44]
	139	cinncassin A_1_	Twig	[44]
Other Compounds	140	benzyl benzoate	Twig	[33]
	141	2-hydroxybenzaldehyde	Twig	[33]
	142	3-phenylpropanol	Twig	[33]
	143	2,2,4,6,6-pentamethylheptane	Bark	[40]
	144	2,5,9-trimethyldecane	Bark	[40]
	145	2-ethyl-5-propylphenol	Bark	[40]
	146	3,4-dimethoxyphenethyl alcohol	Bark	[40]
	147	2,5-dimethylundecane	Bark	[40]
	148	benzaldehyde	Bark, twig	[33,40]
	149	phenylethyl alcohol	Bark, twig	[33,40]
	150	2-methoxyphenylacetone	Bark	[35]
	151	benzenepropanal	Bark, twig	[33,35]
	152	acetophenone	Bark, twig	[33,36]
	153	benzene,1,3-dimethyl	Bark	[36]
	154	styrene	Bark	[36]
	155	1,3-pentanediol,2,2,4-trimethyl	Bark	[36]
	156	decanal	Bark	[36]
	157	dodecane, 2,6,10-trimethyl	Bark	[36]
	158	epicatechin-(4β→8)-epicatechin-(4β→8)-epicatechin	Bark	[51]
	159	rosavin	Bark, twig	[42,47]
	160	coumarin	Bark, twig	[36,49]
	161	dihydromelilotoside	Twig	[42]
	162	methyl dihydromelilotoside	Twig	[42]
	163	evofolin B	Bark, twig	[44,47]
	164	cinnamomoside A	Twig	[44]
	165	cinncassin C	Bark	[46]
	166	cinncassin B	Bark	[46]

**Table 3:** Pharmacological effects of C. cassia.

Effects	Detail	Extracts/Compounds	Concentration/Dose	In Vivo/In Vitro	Ref.
***Anti-tumor Effects***	Lung cancer				
	Inhibiting Nrf2-regulated enzyme activity and Nrf2 expression	procyanidins	Cell lines of A549, 0–300 μg/mL	in vitro	[59]
	Inhibiting Nrf2 expression and cell proliferation	procyanidins	Cell lines of A549, LK-2 and LU-99, 2.5 μg/mL	in vitro	[60]
	Inhibiting Nrf2 expression and activation of IGF-1R phosphorylation	procyanidins	Cell lines of A549, LU-99, 10 μg/mL	in vitro	[61]
	Inhibiting TGF-β-induced EMT	WEBC procyanidin C1	Cell lines of A549, 12.5–200 μg/mL 1.25–40 μg/mL	in vitro	[51]
	Inhibiting TGF-b1-induced EMT	EEC EEC	Cell lines of A549, H1299, 20–60 μg/mL 100,200 mg/Kg	in vitro in vivo	[62]
	Repressing u-PA/MMP-2 via FAK to ERK1/2 pathways	EEC	Cell lines of A549, H1299, 0–60 μg/mL	in vitro	[63]
	Inhibiting the activity of pyruvate dehydrogenase kinase (PDHK)	WETC	Cell lines of A549, H1299 and LLC, 0–200 μg/mL	in vitro	[64]
	Oral cancer				
	Cytotoxic effects on HSC-3 cells	EOC cinnamaldehyde	HSC-3 cell line, 2.5–40 μg/mL, IC_50_ = 13.7 μg/mL 2.5–40 μg/mL, IC_50_ = 10 μg/mL	in vitro	[65]
	Enhancement of autophagy markers to induce cell apoptosis	EETC	0–100 μg/mL 250, 500 mg/Kg	in vitro in vivo	[66]
	Breast cancer				
	Cytotoxic effects on MCF-7 and MDA-MB-231	HEBC	Cell lines of MCF-7 and MDA-MB-231, 50, 100, 200 μg/mL, IC_50_ = 34 μg/mL, IC_50_ = 32.42 μg/mL	in vitro	[67]
	Cervical cancers				
	Inducing cell apoptosis	WEBC	SiHa cell line, 0–80 μg/mL	in vitro	[68]
	Head and neck squamous cell carcinoma (HNSCC)				
	Inhibiting EGFR-TK activity	EOTC	0.625–10 μg/mL	in vivo	[53]
***Anti-Inflammatory and Analgesic Effects***	Inhibiting LPS-stimulated inflammatory and carrageenan induced hind paw edema	cinnamaldehyde	murine macrophage cell line RAW264.7, 6.25–50 μM 1.25, 2.5 and 5 mg/kg	in vitro in vivo	[69]
	Inhibiting LPS-stimulated inflammatory	WEBC	20, 100 and 500 mg/kg peritoneal macrophages, 10, 50, 100, 200, and 400 μg/mL	in vivo in vitro	[4]
	Inhibiting LPS-stimulated inflammatory	EOLC cinnamaldehyde	Macrophage J774A.1 cells, 1.25–20 μg/mL, IC_50_ = 6.1 ± 0.25 μg/mL IC_50_ = 9.97 ± 0.35 μg/mL	in vitro	[34]
	Inhibitory effects on NO production and TNF-α	EAEBC	RAW 264.7 and J774A.1 macrophages, IC_50_ = 19.7 ± 78.4 μg/mL, LC_50_ = 140 ± 9.0 μg/mL	in vitro	[70]
	Inhibiting carrageenan induced hind paw Edema, oxytocin and acetic acid-induced abdominal constriction test	EOTC	15, 30, and 60 mg/kg	in vivo	[52]
	Inhibitory effects on NO production	EAEBC	BV-2 cells	in vitro	[46]
	Inhibiting LPS-induced septic shock and inflammasome	EEBC	25, 50 and 100 mg/kg	in vivo	[71]
	Inhibiting matrix metalloproteinases	cinnamomulactone	FLS cells, 0.1, 1, 10 and 100 μM	in vitro	[49]
	Inhibiting Complete Freund’s adjuvant (CFA)-induced arthritis	WEBC	50, 100 and 200 mg/kg	in vivo	[72]
	Inhibitory effects against Oxaliplatin-Induced Neuropathic Cold Allodynia	WEBC coumarin	100, 200 and 400 mg/kg 10 mg/kg	in vivo	[74]
	Inhibitory effects against oxaliplatin-induced neuropathic cold allodynia	cinnamic acid	10, 20 and 40 mg/kg	in vivo	[75]
***Anti-diabetic and obesity effect***	Exhibiting potent hypoglycemic activity	WEBC	100, 250 and 500 mg/kg insulinoma RINm5F cells, 0.125, 0.25, 0.5 and 1.0 mg/mL	in vivo in vitro	[3]
	Exhibiting potent hypoglycemic activity	polyphenols	10 and 50 mg/kg	in vivo	[76]
	Inhibiting α-glucosidase, Sucrase and Maltase	AEBC	IC_50_ = 0.474 mg/mL, IC_50_ = 0.10 mg/mL, IC_50_ = 0.38 mg/mL	in vitro	[77]
	Exhibiting potent hypoglycemic activity	de-coumarinated extracts	200 mg/kg	in vivo	[78]
	Inhibiting diabetic nephropathy	EEBC	Rat mesangial cells, 10 μM	in vitro	[47]
	Inhibiting diabetic nephropathy	EEBC	Rat mesangial cells, 10, 30 and 50 μg/mL	in vitro	[37]
	Preventing chronic complications of experimentally induced type II diabetes	EBC	500, 1000 and 1500 mg/kg	in vivo	[79]
	Protecting diabetic kidney	CcAgNPS	5, 10 and 200 mg/kg	in vivo	[80]
	Preventing of obesity	EC	3T3-L1 cell, 50, 100 and 200 μg/mL	in vitro	[81]
	Preventing high-fat diet-induced obesity	WEBC	100, 300 mg/kg C2C12 myoblasts, 0.1, 0.2 mg/mL	in vivo in vitro	[82]
***Antibacterial and Antiviral Effects***	Inhibitory effects against *Staphylococcus aureus, Aspergillus niger, Bacillus subtilis* and *Escherichia coli*	EOBC	MIC = 200, 200, 200 and 400 μg/mL	in vitro	[54]
	Inhibitory effects against *Escherichia coli, Staphylococcus aureus, Klebsiella pneumoniae* and *Pseudomonas aeruginosa*	EOBC	MIC = 0.28, 0.28, 0.56 and 0.11 mg/mL	in vitro	[83]
	Inhibitory effects against non-O157 STECs	EOBC	MIC = 0.025% (*v*/*v*)	in vitro	[84]
	Inhibitory effects against E*scherichia coli* and *Staphylococcus*	EOBC	MIC = 1.0 mg/mL	in vitro	[85]
	Inhibitory effect against *Staphylococcus hyicus* and *Staphylococcus aureus*	EOC	MIC = 0.078%	in vitro	[86]
	Inhibitory effects against *propionibacterium acnes*, *Staphylococcus Epidermidis* and *Staphylococcus aureus*	EOBC	MIC = 0.156, 0.313 and 0.25 μL/mL	in vitro	[87]
	Inhibitory effect against *Staphylococcus aureus*	EOC	MIC = 500 μL/L	in vitro	[88]
	Inhibitory effects against *Escherichia coli*, *Staphylococcus aureus,* and *Pseudomonas aeruginosa*	EOBC	MIC = 4.88, 4.88, and 19.53 μg/mL	in vitro	[89]
	Inhibitory effects against avian influenza virus subtype H7N3	CcAgNPS	IC_50_ = 125 μg/mL	in vitro	[90]
***Cardiovascular Protective Effects***	Inhibiting proliferation of vascular smooth muscle cells	WEBC	Rat aortic VSMCs, 10, 30 and 50 μg/mL	in vitro	[91]
	Inhibiting angiogenesis	WEC	HUVECs, 0–100 μg/mL	in vitro	[92]
	Preventing diabetic cardiomyopathy	WEBC	750 mg/kg	in vivo	[93]
***Cytoprotective Effects***	Protecting against gastric ulcers induced by stress, ethanol or HCl	CP	2, 10 and 100 mg/g per feed	in vivo	[94]
	Ameliorating cisplatin-induced cytotoxicity	WEBC	Vero cell line, 10–50 μg/mL	in vitro	[95]
***Neuroprotective Effects***	Regulating the 5-HT1A and GABAergic system	EEC	In the acute experiment, 250, 500 and 750 mg/mL In the chronic experiment, 50, 75 and 100 mg/mL	in vivo	[96]
	Region specific change of 5-HT1A receptors	EEBC	100, 750 mg/mL	in vivo	[97]
	Correct cognitive impairment	WEC	0.75 mg/mL PC12 cell line, 1, 10 and 100 μg/mL, IC_50_ = 0.7 μg/mL	in vivo in vitro	[98]
	Neuroprotective effect	total flavonoids	PC12 cell line 20–100 μg/mL	in vitro	[99]
	Inhibiting serotonin reuptake	EC	25 and 50 mg/kg	in vivo	[100]
***Immunoregulation Effects***	Inhibitory effects against proliferation of T cell and B cell	EEBC cinncassiol G and cinnacasol	100 μg/mL 50 and 100 μM	in vitro	[39]
	Inhibitory effects against proliferation of T cell	phenolic glycosides	12.5–200 μM	in vitro	[48]
	Inhibitory effects against proliferation of T cell and B cell	cinnacasside F	400 μM	in vitro	[45]
***Anti-tyrosinase Activity***	Inhibitory effects against tyrosinase	EOBC *trans*-cinnamaldehyde	IC_50_ = 6.16 ± 0.04 mg/mL IC_50_ = 4.04 ± 0.08 mg/mL	in vitro	[40]
	Inhibitory effects against tyrosinase	EOC *trans*-cinnamaldehyde	B16 melanoma cells, 1.0, 2.0, 2.5 and 5.0 μg/mL, IC_50_ = 6.16 ± 0.04 mg/mL 1.0, 2.0 and 2.5 μg/mL, IC_50_ = 4.04 ± 0.08 mg/mL	in vitro	[101]
***Other Pharmacological Effects***	Inhibitory effects against xanthine oxidase	ETC	IC_50_ = 7.8–36.3 μg/mL	in vitro	[42]
	Improving sexual function in young male rats	MEC	0.1, 1, 10, and 100 μg/mL, IC_50_ = 61.72 ± 2.20 μg/mL 100 mg/kg	in vitro in vivo	[102]
	Ameliorating hepatotoxicity	MEBC	50, 100 and 150 mg/kg	in vivo	[103]
	Inhibiting spontaneous uterus contractions	EOTC	Myometrial cells, 15, 30 and 60 mg/kg 25, 50 and 100 μg/mL, IC_50_ = 361.3 μg/mL	in vivo in vitro	[104]
	Inhibitory effect against *Leishmania donovani* infection	EBC	Peritoneal macrophages, IC_50_ = 33.66 ± 3.25 μg/mL 50 and 100 mg/kg	in vitro in vivo	[107]

## Abbreviations

HUVECs	human umbilical vein endothelial cells
WEC	water extracts of *C. cassia*
EEC	ethanol extracts of *C. cassia*
EC	extracts of *C. cassia*
EBC	extracts of barks of *C. cassia*
ETC	extracts of twigs of *C. cassia*
WEBC	water extracts of barks of *C. cassia*
EEBC	ethanol extracts of barks of *C. cassia*
MEBC	methanol extracts of barks of *C. cassia*
WETC	water extracts of twigs of *C. cassia*
EETC	ethanol extracts of twigs of *C. cassia*
EOBC	essential oil of barks of *C. cassia*
EOTC	essential oil of twigs of *C. cassia*
EOC	essential oil of *C. cassia*
EOLC	essential oil of leaves of *C. cassia*
HEBC	hexane extracts of barks of *C. cassia*
EAEBC	ethyl acetate extracts of barks of *C. cassia*
AEBC	acetone extract of barks of *C. cassia*
CcAgNPS	*C. cassia* silver nanoparticles
CP	*C. cassia* powder
MEC	methanol extracts of *C. cassia*
